# PITX1 Is a Regulator of TERT Expression in Prostate Cancer with Prognostic Power

**DOI:** 10.3390/cancers14051267

**Published:** 2022-03-01

**Authors:** Alexandra M. Poos, Cornelia Schroeder, Neeraja Jaishankar, Daniela Röll, Marcus Oswald, Jan Meiners, Delia M. Braun, Caroline Knotz, Lukas Frank, Manuel Gunkel, Roman Spilger, Thomas Wollmann, Adam Polonski, Georgia Makrypidi-Fraune, Christoph Fraune, Markus Graefen, Inn Chung, Alexander Stenzel, Holger Erfle, Karl Rohr, Aria Baniahmad, Guido Sauter, Karsten Rippe, Ronald Simon, Rainer Koenig

**Affiliations:** 1 Integrated Research and Treatment Center, Center for Sepsis Control and Care (CSCC), Jena University Hospital, Am Klinikum 1, 07747 Jena, Germany; a.poos@dkfz-heidelberg.de; 2 Division of Chromatin Networks, German Cancer Research Center (DKFZ) and BioQuant Center, Im Neuenheimer Feld 280, 69120 Heidelberg, Germany; d.braun@dkfz-heidelberg.de (D.M.B.); caroline.knotz@dkfz-heidelberg.de (C.K.); l.frank@dkfz-heidelberg.de (L.F.); inn.chung@med.uni-heidelberg.de (I.C.); karsten.rippe@dkfz-heidelberg.de (K.R.); 3 Department of Pathology, University Medical Center Hamburg-Eppendorf, Martinistraße 52, 20251 Hamburg, Germany; cor.schroeder@uke.de (C.S.); j.meiners@uke.de (J.M.); a.polonski@uke.de (A.P.); g.makrypidi@uke.de (G.M.-F.); c.fraune@uke.de (C.F.); g.sauter@uke.d (G.S.); r.simon@uke.de (R.S.); 4 Institute for Infectious Diseases and Infection Control (IIMK), Jena University Hospital, Am Klinikum 1, 07747 Jena, Germany; neeraja.jaishankar@uni-jena.de (N.J.); daniela.roell@med.uni-jena.de (D.R.); marcus.oswald@web.de (M.O.); 5 Institute of Human Genetics, Jena University Hospital, Am Klinikum 1, 07747 Jena, Germany; alexander.stenzel@med.uni-jena.de (A.S.); aria.baniahmad@med.uni-jena.de (A.B.); 6 VIROQUANT CellNetworks RNAi Screening Facility and Research Group High-Content Analysis of the Cell (HiCell), BioQuant Center, Heidelberg University, Im Neuenheimer Feld 267, 69120 Heidelberg, Germany; manuel.gunkel@bioquant.uni-heidelberg.de (M.G.); holger.erfle@bioquant.uni-heidelberg.de (H.E.); 7 Biomedical Computer Vision Group, BioQuant Center and IPMB, Heidelberg University and German Cancer Research Center (DKFZ), Im Neuenheimer Feld 267, 69120 Heidelberg, Germany; roman.spilger@bioquant.uni-heidelberg.de (R.S.); thomas.wollmann@bioquant.uni-heidelberg.de (T.W.); k.rohr@dkfz-heidelberg.de (K.R.); 8 Martini-Clinic, Prostate Cancer Center, University Medical Center Hamburg-Eppendorf, 20251 Hamburg, Germany; graefen@uke.de

**Keywords:** regulatory networks, prostate cancer, biomarkers, PITX1, mixed integer linear programming, modularity, transcription factors

## Abstract

**Simple Summary:**

Most prostate cancer is of an indolent form and is curable. However, some prostate cancer belongs to rather aggressive subtypes leading to metastasis and death, and immediate therapy is mandatory. However, for these, the therapeutic options are highly invasive, such as radical prostatectomy, radiation or brachytherapy. Hence, a precise diagnosis of these tumor subtypes is needed, and the thus far applied diagnostic means are insufficient for this. Besides this, for their endless cell divisions, prostate cancer cells need the enzyme telomerase to elongate their telomeres (chromatin endings). In this study, we developed a gene regulatory model based on large data from transcription profiles from prostate cancer and chromatin-immuno-precipitation studies. We identified the developmental regulator PITX1 regulating telomerase. Besides observing experimental evidence of PITX1′s functional role in telomerase regulation, we also found PITX1 serving as a prognostic marker, as concluded from an analysis of more than 15,000 prostate cancer samples.

**Abstract:**

The current risk stratification in prostate cancer (PCa) is frequently insufficient to adequately predict disease development and outcome. One hallmark of cancer is telomere maintenance. For telomere maintenance, PCa cells exclusively employ telomerase, making it essential for this cancer entity. However, TERT, the catalytic protein component of the reverse transcriptase telomerase, itself does not suit as a prognostic marker for prostate cancer as it is rather low expressed. We investigated if, instead of *TERT*, transcription factors regulating *TERT* may suit as prognostic markers. To identify transcription factors regulating *TERT*, we developed and applied a new gene regulatory modeling strategy to a comprehensive transcriptome dataset of 445 primary PCa. Six transcription factors were predicted as *TERT* regulators, and most prominently, the developmental morphogenic factor PITX1. PITX1 expression positively correlated with telomere staining intensity in PCa tumor samples. Functional assays and chromatin immune-precipitation showed that PITX1 activates *TERT* expression in PCa cells. Clinically, we observed that PITX1 is an excellent prognostic marker, as concluded from an analysis of more than 15,000 PCa samples. PITX1 expression in tumor samples associated with (i) increased Ki67 expression indicating increased tumor growth, (ii) a worse prognosis, and (iii) correlated with telomere length.

## 1. Introduction

Prostate cancer (PCa) shows the second-highest incidence of cancer in men and is the fifth most frequent leading cause of cancer death [1]. Established screening of the prostate-specific antigen (PSA) level improved early diagnosis, and nearly 90% of PCa can be localized clinically at the time of their diagnosis [2]. Even though most of the patients have an indolent form of PCa and are curable, some PCa belongs to rather aggressive subtypes leading to metastasis and death [3,4]. Therapeutic options are radical prostatectomy, radiation or brachytherapy, and, in some cases, also active surveillance or applying androgen depletion therapy. For patients with metastasis, drug treatment is necessary by applying chemotherapeutics (docetaxel, cabazitaxel) or androgen receptor inhibitors (e.g., enzalutamid) [5]. Of overriding importance for the appropriate treatment decision, well-established risk stratification is necessary. Until now, the suggested risk stratification combines Gleason score, pre-operative PSA-levels in the blood, and further pathological and clinical staging. However, these measurements are insufficient to adequately predict the outcome of patients [6], making a surveillance strategy hazardous, particularly if the prediction needs to be made before prostatectomy. On top of PSA, complementary molecular biomarkers may improve stratifying the risk of death and progression of the disease. Previous studies of primary PCa identified several recurrent genomic alterations such as mutations, gene fusions, DNA copy-number changes, and rearrangements. The most common alteration is the fusion of the genes *TMPRSS2* and *ERG* [7]. *SPOP*, *TP53*, *FOXA1,* and *PTEN* are the most frequently mutated genes [8]. Based on gene fusions, mutations (mainly ETS), epigenetic changes (e.g., DNA methylation changes upon IDH1 mutation), and androgen receptor (AR) activity, seven subtypes of primary PCa were specified by The Cancer Genome Atlas Research Network [3,4]. However, such genetic subtyping does not lead to a mechanistic understanding of the patho-mechanisms. Here, we improved risk stratification in PCa by identifying new biomarkers via a mechanism-based approach investigating the regulation of telomerase.

Telomere length maintenance is one of the hallmarks of cancer needed for replicative immortality of cancer cells [9]. Telomeres are nucleoprotein structures at the ends of eukaryotic chromosomes, protecting them against fusion, degradation, and unwanted activation of double-strand break repair mechanisms [10,11]. Telomeres progressively shorten with each cell division. In somatic cells, after a limited number of replications, this can induce replicative senescence or apoptosis, thereby acting as a barrier to unlimited proliferation and tumorigenesis [12]. Cancer cells overcome this restriction and maintain their telomeres by re-expressing *TERT*, the catalytic protein component of telomerase. For several tumor entities, also alternative telomere maintenance mechanisms have been described in which *TERT* is not involved (ALT, Alternative Lengenthing of Telomeres), but these mechanisms have not been observed in PCa [13]. *TERT* is usually not expressed in differentiated somatic cells. It gets reactivated in tumor cells to extend the telomeric repeats [14,15]. Although *TERT* is central for immortality, it typically has very low expression in PCa cells, making it difficult to use it as a prognostic marker. Accordingly, we aimed at identifying regulators (transcription factors, TF) of *TERT,* which may better suit as biomarkers for risk stratification. *TERT* obeys a fine-grained and balanced regulation [16]. Hence, we studied its regulation by investigating a gene regulatory network model. In contrast to previous modeling concepts of us and others, we studied not only the role of directly acting TF, but also TF indirectly regulating *TERT* expression. For incorporating indirectly regulating TF, we integrated the modularity framework from graph theory into our previously developed regulatory interaction predictor (MIPRIP, see [17]). In graph theory, a scientific aim is to detect communities in a graph or network, which are either cliques or more relaxed clusters of highly connected nodes (for us, the genes) in the network. Here, nodes are grouped into subsets such that their interactions are dense within the subset but sparse between the subsets [18,19,20,21]. Interestingly, not all networks are suitable for such divisions making the detection of such dense groups likely to have a semantic structure [22]. A semantic structure in our application would be a regulatory subnetwork being specific for a certain disease, malignancy, or other condition. Here, we focussed on identifying a semantic structure comprising direct regulators and regulators of *TERT*. Using the modularity approach, originally developed by Newman and coworkers [23], we assumed that direct regulators of *TERT* are densely intertwined with their direct regulators. Identifying such a module would support a mechanistic understanding of the specific *TERT* expression in subsets of PCa, and may serve as a solid basis for prognostic biomarker predictions.

Using a comprehensive set of transcription profiles from PCa samples, we employed our gene regulatory model MIPRIP yielding directly acting TF of telomerase (in the following denoted as “direct regulators”), together with an integrated modularity-based model inferring indirectly acting TF (“indirect regulators”).

## 2. Materials and Methods

The data preprocessing, model implementation and all statistical analysis were performed using R version 3.5.1 (www.r-project.org, accessed on 15 July 2018). To solve the MILP optimization problems Gurobi Optimizer version 7.0.1 was used under an academic license. The visualization of the network was conducted using Cytoscape version 3.6.1.

### 2.1. Gene Expression Data

Publicly available RNA-Seq data of 497 PCa patients and 52 healthy prostate tissue samples of The Cancer Genome Atlas (TCGA) was downloaded from GDAC of the Broad Institute (http://gdac.broadinstitute.org/, release 28 January 2016, accessed on 31 March 2016). For our network model, we used the normalized and log2 transformed RSEM values. All genes with more than 25% NAs and low varying genes (standard deviation SD ≤ 0.5) were filtered out. Then, a z-score transformation for each gene across the whole dataset was performed. For modeling, we removed all samples with no *TERT* expression value. This resulted in 445 PCa and 18 healthy prostate tissue samples.

### 2.2. The Mixed Integer Linear Model of TERT Regulation

We used our previously developed “Mixed Integer linear Programming based Regulatory Interaction Predictor” (MIPRIP) tool [17,24] (version 2, dual-mode) to study the regulation of the telomerase reverse transcriptase (*TERT*) gene in PCa versus healthy prostate tissue. MIPRIP is a software package for R (www.r-project.org) and is freely available at https://github.com/KoenigLabNM/MIPRIP. The basic principle of MIPRIP is that the expression of the gene of interest is predicted by the activity of the regulators potentially binding to the gene’s promoter. Due to post-translational modifications, protein stability, and other effects, the activity of a transcription factor (TF) depend only partially on the gene expression of the TF itself. Hence, we and others inferred the activity of a TF by the expression of its potential target genes [25,26,27]. We used our previously constructed generic network of the regulator to target gene interactions. These interactions were derived from promoter binding information from 7 different resources reporting mainly the results from ChIP-experiments. In summary, for the generic network we integrated interactions from MetaCore^TM^ (https://portal.genego.com/, accessed on 24 February 2022), ChEA [28], Encode [29], HmCHIP [30], HTRI [31], ChIPBase [32] and a motif analysis using the total binding affinity (TBA) [33,34]. The information of these resources was weighted based on the reliability of the source, and the weights were added if the interaction was found in more than one source [17,24]. The predicted gene expression value ${\tilde{g}}_{ik}\;$ of the *TERT* gene was calculated as:

(1) $${\tilde{g}}_{ik}\;=\;\beta_{0}\;+\;\sum_{t\;=\;1}^{T}\beta_{t}*\;es_{ti}*\;act_{tk}$$

where *β*_0_ is an additive offset, *T* the number of all investigated regulators, *β_t_* the optimization parameter for regulator *t*, $es_{ti}$ the edge score between regulator *t* and its putative target gene *i* based on the generic gene regulatory network and $act_{tk}$ the activity of regulator *t* in sample *k*. The activity value of a regulator was defined as the cumulative effect of a regulator on all its target genes and was calculated as

(2) $$act_{tk}\;=\;\frac{\sum_{i\;=\;1}^{n}es_{ti}\;*\;g_{ik}}{\sum_{i\;=\;1}^{n}es_{ti}}$$

$act_{tk}$ is the estimated effect of regulator *t* in sample *k*, $es_{ti}$ the edge score between regulator *t,* and gene *i*, $g_{ik}$ the gene expression of gene *i* in sample *k*. For the activity calculation, the gene expression value of the target gene (*TERT*) itself was excluded. The edge score $es_{ti}\;$ was the edge weight between the regulators and the target genes. If gene *i* was reported to be a target of regulator *t* the edge weight was higher than 0.

To solve this optimization problem, we used the optimizer Gurobi (www.gurobi.com, accessed on 20 March 2017). To gain a representative variety of models with different sizes, we constructed models with one up to 10 regulators and performed 10-times threefold cross-validation to avoid overfitting. For this, the dataset was 10-times randomly divided into thirds, where two-thirds were used to identify the best TF combination and the remaining third to predict the gene expression of *TERT* with this TF combination. The performance of the model was determined by the correlation between the measured and the predicted gene expression value.

### 2.3. Regulatory TF-TF Network

To construct a regulatory TF-TF network, MIPRIP was combined with the concept of modularity from Newman [23]. With this approach, we aimed to find a highly connected module consisting of direct and indirect regulators $R_{t}$ of our particular gene *TERT*. Indirect regulators were direct regulators of the direct regulators of *TERT*. In the following, all regulators binding to the promoter of the particular gene were called direct, while the regulators of the regulators were called indirect regulators of the particular gene. The MILP was as follows:

(3) $$Maximize\;\sum_{t_{1},t_{2}\in \;V;\;t_{1}\;\neq \;t_{2}}w_{t_{1}t_{2}}^{\prime }\;\cdot \;y_{t_{1}t_{2}}$$

(4) $$Subject\;to\;x_{t_{1}}\;+\;x_{t_{2}}\;-\;y_{t_{1}t_{2}}\leq 1$$

(5) $$y_{t_{1}t_{2}}\;\leq \;x_{t_{1}}$$

(6) $$y_{t_{1}t_{2}}\;\leq \;x_{t_{2}}$$

(7) $$\sum_{t\;=\;1}^{T}x_{t}\;\leq \;limit$$

(8) $$w_{t_{1}t_{2}}\;=\;cor\left(act_{t_{1}k},act_{t_{2}k}\right)\;\cdot \;es_{t_{1}t_{2}}$$

(9) $${\tilde{w}}_{t_{1}t_{2}}\;=\;w_{t_{1}t_{2}}\;-\;\frac{d_{t_{1}}\;d_{t_{2}}}{2m}$$

with $d_{t}\;=\;\sum w_{t_{1},t_{2}}$ and $m\;=\;\frac{1}{2}\sum_{t\;=\;1}^{T}d_{t}$

(10) $$x_{t}\in \left\{0,1\right\}\;,\;y_{t_{1}t_{2}}\in \left\{0,1\right\},$$

where *t* indicates the nodes (regulators), *w* are the edge weights, *d* the degree of the node and *T* the number of all regulators. *x* and *y* are binary parameters and indicate if the nodes and edges were selected. The objective of the modularity-based MILP approach was to maximize the sum of edge weights between the connected nodes in the modularity network (Formula (3)). Constraint (4) enforces that if node *t_1_* and node *t_2_* were in the module, then also the edge between *t_1_* and *t_2_* had to be in the module. By constraints (5) and (6) it was ensured that only edges were selected for which both end nodes were inside the module. The size *T* of the module was constrained by Equation (7). The goal of the modularity was to identify a highly connected module which can best explain the regulation of the particular gene of interest. Therefore, the sum of the edge weights between the connected nodes inside the modules was maximized and penalized if their end nodes had high degrees. The corresponding edge weights *w* were computed as described in (8) and (9) by multiplying the correlation of each regulator pair’s activity over all investigated samples *k* with the corresponding edge weights in the generic network. Because this weight was not always the same between node (regulator) *t_1_* and *t_2_*, the mean value of both directions was taken. All these weights were computed in a preprocessing step and were constants in the MILP. For the combined model of MIPRIP and modularity, all equations of MIPRIP [17,24] and the equations above were used. As objective function of the combined model, the sum of objective functions of the single models was used:

(11) $$Minimize\;\sum_{k\;=\;1}^{l}e_{i,k}\;-\;\lambda \;\sum_{t_{1},t_{2}\in \;V;\;t_{1}\neq t_{2}}w_{t_{1}t_{2}}^{\prime }\;\cdot \;y_{t_{1}t_{2}}.$$

MIPRIP    Modularity

Variables of the direct regulators were the same for both optimization parts. The parameter λ controlled the tradeoff between MIPRIP and the modularity network (Equation (11)). The performance of the model was determined as with MIPRIP alone. The best subnetwork consists of the combination of MIPRIP regulators (defined by the $x_{t}$), which was used most often in all models and the corresponding modularity regulators.

**Figure 1:**
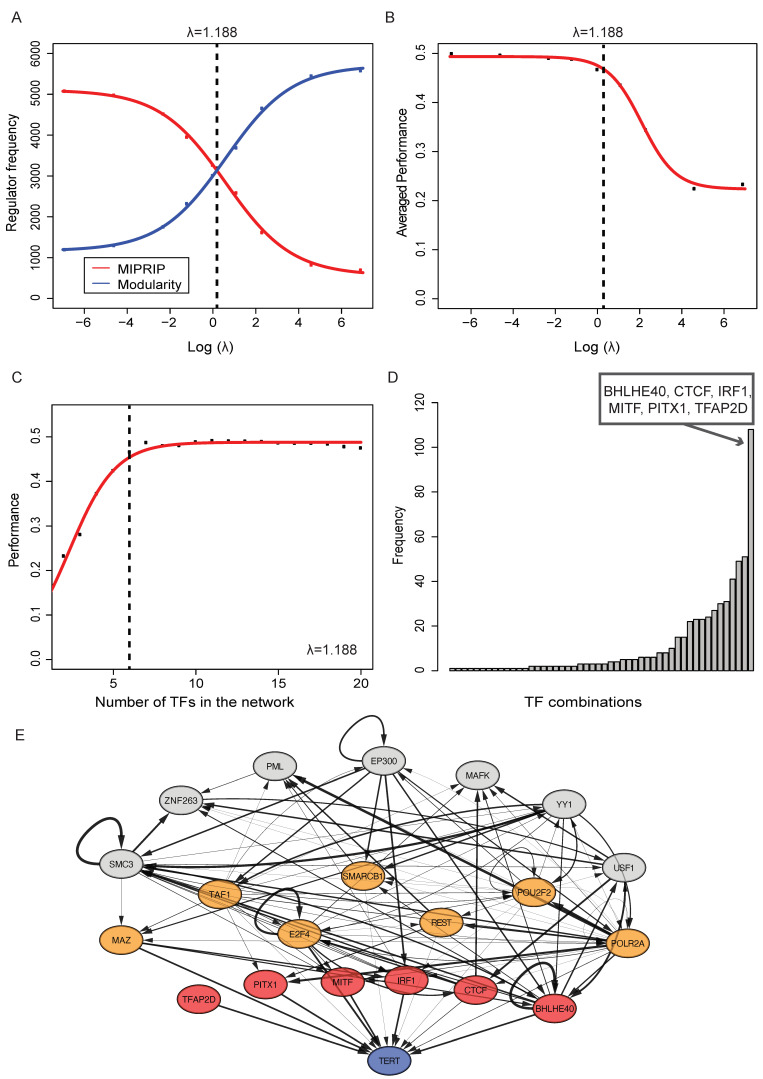
Optimization of λ and the identified regulatory module. (**A**) The sum of selected TF from the MIPRIP model (direct TF, red curve) and from the modularity model (indirect TF, blue curve) over all models for different λ values. The total number of direct and indirect TF to be selected by the models was 6270 for each λ value (from 30 repeated cross-validations, in which each repeat consisted of models from 2 to 20 TF). The intersection of both curves led to the optimal λ value. (**B**) The performance over all models for different λ values. The dashed line indicates the value for the optimal λ. (**C**) Shown is the performance of the models with the optimal λ. At least six TF are necessary to obtain a good prediction of *TERT* expression. (**D**) This histogram shows which combination of TF was used most often over all models. The most often combination was BHLHE40, CTCF, IRF1, MITF, PITX1, and TFAP2D. (**E**) The identified gene regulatory network for *TERT* regulation in PCa, predicted direct regulators of *TERT* are marked in red. TF added by the modularity approach are marked in orange if they were known to bind to the *TERT* promoter and in grey if they only bind to the indirect regulators of *TERT*. The width of the edges between the significant and putative regulators and *TERT* is based on their weights in the generic regulatory network. The width of the interactions between the regulators was derived by the correlation of their activity values multiplied with the weights in the generic regulatory network.

In this study, this combined model was used to identify the regulatory subnetwork best explaining the regulation of *TERT*. To reduce computational complexity, we run MIPRIP (single-mode) first for the identified significant direct regulators of *TERT* (Table 1) and continued only with regulators, which were used in at least 20% of the models (Table 2). Because the gene expression data of CTCF and NR2F2 was filtered out based on low variances, the unfiltered gene expression values of these 2 genes were used to construct a MIPRIP model. For TFAP2D, nearly all gene expression values were NA, and because of that, no MIPRIP model was possible. Because the combined model is only based on regulator activity values, for TFAP2D no further regulators were added to the restricted list of indirect regulators. These preprocessing steps resulted in 12 direct regulators and 72 indirect regulators for the combined approach. We constructed combined models of 2 up to 20 direct and indirect regulators. To optimize the parameter λ, models with different sizes of λ (0.001, 0.01, 0.1, 0.3, 1, 3, 10, 100, 1000) were constructed, and the optimal tradeoff between the number of direct and indirect regulators was estimated by counting how many direct and indirect regulators were selected. These counts of the MIPRIP (red curve Figure 1A) and the modularity (blue curve) part of the model were plotted for the 9 different λ values. The intersection of both curves indicated the optimal λ balancing the number of direct and indirect regulators over all models. Furthermore, good performance of *TERT* prediction was observed (Figure 1). As for the MIPRIP model alone, also for the combined model, 10-times three-fold cross-validation was performed.

### 2.4. Immunohistochemical Analysis of PITX1 and IRF1 in PCa Patients

The immunohistochemistry (IHC) staining of PITX1 and IRF1 was performed on a freshly cut tissue microarray of 17,747 patients on one day and in one experiment (separately for PITX1 and IRF1). Patients were collected between 1992 and 2014 at the University Medical Center Hamburg-Eppendorf (Department of Urology and the Martini Clinics), and all patients underwent radical prostatectomy (RPE). The analysis of the RPE specimens was performed as described in [35]. For all patients, histopathological data such as tumor state, Gleason grade, nodal stage, and stage of the resections were available, as well as for 14,464 patients also follow-up data (1 to 275 months; 48 months median). For a subset of patients there was also information on ERG expression (*n* = 10,677) [36], 10q23 (*PTEN*) deletion (*n* = 6704) [37], 3p14 (FOXP1) deletion (*n* = 7201) (expanded from [38]), 6q15 (MAP3K7) deletion (*n* = 6069) (expanded from [39]) and 5q21 (CHD1) deletion (*n* = 8074) (expanded from [40]) present. The prostate-specific antigen (PSA) levels were controlled, post-RPE and PSA recurrence was defined as a PSA level of ≥ 0.2 ng/mL or an increasing PSA level in subsequent measurements. The manufacturing process of TMA was performed as described in [41,42], and each TMA block also contained controls, e.g., normal prostate tissue. For the staining, the slides were first deparaffinized and exposed to heat-induced antigen retrieval for 5 min in an autoclave at 121 °C in pH 7.8 Tris-EDTA-Citrate buffer. The primary PITX1 antibody HPA008743 (Sigma, polyclonal rabbit, dilution: 1:59) was utilized for 60 min at 37 °C. After incubation, the bound antibody was visualized with the EnVision Kit (Dako, Glostrup, Denmark) according to the manufacturer’s directions. The IHC staining of PITX1 was then validated with positive and negative control tissues on the TMA and was in line with data from the Human Protein Atlas [43]. Glandular cells of the normal prostate showed no PITX1 expression (data not shown). Tumor samples with no staining intensity were scored as “negative” (Figure S1A), while a staining intensity of 1+, or 2+ in >30% and ≤ 70% of tumor cells, or 3+ in ≤30% of tumor cells (Figure S1B) were classified as “low” and samples with a staining intensity of 2+ in >70% of tumor cells or 3+ in >30% of tumor cells as “high” (Figure S1C). Some spots were not evaluable because of lack of tissue samples or absence of unequivocal cancer tissue in the TMA spot.

All statistical analyses were performed using JMP version 12.0 (SAS Institute Inc., Cary, NC, USA). To identify associations between PITX1 expression and the clinico-pathological variables, contingency tables were calculated and tested for significance based on a chi-square test. For the Kaplan–Meier curves, the PSA-recurrence free survival was used as a clinical endpoint. Significant differences in survival between the stratified patient cohorts (high, low and negative PITX1 expression) were identified on a log-rank test. Using a Cox proportional hazards regression analysis, statistical independence and significance between the clinical, pathological, and molecular variables were tested.

The analysis of IRF1 (rabbit monoclonal antibody, Abcam, ab186384; dilution 1:450), TFAP2D [44], and CTCF [45] was performed similarly as described for PITX1. IRF-1 positive staining was usually seen in all tumor cells (100%). Therefore, the staining intensity was estimated in 4 categories, i.e., negative (not detectable), weak, moderate, and strong staining intensity. For statistical analyses, IRF-1 staining was grouped in low (including negative, weak, and moderate staining) and high (including strong staining).

All archived diagnostic leftover tissues were pseudo-anonymized and used for research purposes without consent as approved by local laws (HmbKHG, §12a) and by the local ethics committee (Ethics commission Hamburg, WF-049/09). The study was conducted in compliance with the Helsinki Declaration.

### 2.5. Cell Lines

PCa cell lines were cultured in their respective growth medium up to a maximum of 80% confluence in a CO_2_ incubator (5%). Respective Cell line/medium pairs were: LNCaP-tet (received 2003) in RPMI 1640 supplement with 10% FCS, 25 mM HEPES (pH 7.5), penicillin (100 U/mL), streptomycin (100 U/mL); C4-2 (received 2000) in DMEM containing phenol red supplemented with 5% FCS, 25 mM HEPES (pH 7.5), penicillin (100 U/mL), streptomycin (100 U/mL), 20% F-12 nutrient mix; PC3 (received 2004) and PC3-AR (received 2004) in RPMI 1640 supplement with 10% heat inactivated FCS, 25 mM HEPES (pH 7.5), penicillin (100 U/mL), streptomycin (100 U/mL) [46,47,48].

### 2.6. siRNA Knockdown

siRNA knock down was performed using ON-TARGETplus siRNA Reagents and the respective ON-Target Plus SMARTPool siRNA products (including non-targeting control Pool) all from Dharmacon. Briefly, 300,000 cells were seeded per 6 well-plate in 2 mL of the respective medium without antibiotics and incubated overnight. The following day medium was refreshed 1 h before transfection, and the transfection was conducted according to manufacturer’s protocol. To notice, DharmaFECT Reagent 1 was used for LNCaP and PC3-AR and Reagent 3 for C4–2 and PC3.

Western blotting was performed 72 h post-transfection using the following antibodies: anti-PITX1 (ab70273, Abcam), anti-α-Tubulin (sc-2005, Santa Cruz), anti-rabbit IgG HPR (sc-2370, Santa Cruz). Densitometric analyses were performed using LabImage.

Total RNA extraction was performed 48 h post-transfection by combining two 6 wells per condition, using Trifast (Peqlab, Radnor, PA, USA), and following the manufacturer´s protocol. For qRT-PCR the two-step method was used, performing the cDNA synthesis with the High Capacity cDNA Reverse Transcription kit (Thermo Fisher, Waltham, MA, USA) and quantitative reverse transcription with the SsoAdvanced Universal SYBR Green Supermix (Bio-rad, Hercules, CA, USA). The following primers were used for the respective mRNA: *hTERT* forward: CGGAAGAGTGTCTGGAGCAA, reverse: GGATGAAGCGGAGTCTGGA; *TBP* forward: GGCGTGTGAAGATAACCCAAGG, reverse: CGCTGGAACTCGTCTCACT; GAPDH forward: AGTCCCTGCCACACTCAG, reverse: TACTTTATTGATGGTACATGACAAGG; *Tubulin* forward: TGGAACCCACAGTCATTGATGA, reverse: TGATCTCCTTGCCAATGGTGTA.

### 2.7. Chromatin Immunoprecipitation

ChIP was performed 72 h post transfection according to the manufacturer´s protocol using the SimpleChIP enzymatic chromatin IP kit (magnetic beads)(cell signaling technology). Transfection was adapted for 15 cm cell culture dishes and seeding 4 × 10^6^ cells per dish. For IP the same PITX1 antibody as for Western Blot was used. qPCR for the *hTERT* promoter region was performed using the SsoAdvanced Universal SYBR Green Supermix (Bio-rad and the following primers obtained from Qi et al. [49]. −1.3kb forward TTTCCAAACCGCCCCTTT, reverse CTGTCACGCTCGCTGGAG. As negative control primers for the -0.1kb TERT promoter region were used to show specific PITX1 binding and antibody functionality, forward: TGCCCCTTCACCTTCCAG, reverse: GCGCTGCCTGAAACTCGC.

### 2.8. Statistical Analysis

For statistical analysis, a two-tailed unpaired student t-test was used. The *p*-value ≤0.05 was considered as statistically significant (*p* ≤ 0.05 *, *p* ≤ 0.01 **, *p* ≤ 0.001 *** and *p* ≤ 0.0001 ****).

## 3. Results

### 3.1. The Direct Model Predicts Specific Transcription Factors of TERT in PCa

First, we identified TF directly regulating *TERT*. 75 TF known to bind at the *TERT* promoter were selected from databases storing experimentally derived binding data (ChIP, ChIP-seq, and ChIP-chip) and computationally inferred (motif-based) TF-binding predictions. Then, we constructed gene regulatory regression models using MIPRIP to fit gene expression values of *TERT* across all tumor and all healthy control samples based on the activities of these 75 potential regulators. We constructed a large set of models using subsets of transcription profiles from the prostate tumor or tissue samples of normal prostate (*n* = 445 tumor and *n* = 18 healthy control samples). Comparing the TF frequencies in the models of tumors and healthy controls led to a list of 17 significant TF predicted to regulate *TERT* specifically in prostate tumors (Table 1). Table S1 in the Supplementary Material also lists 40 TF, which were significant for the controls. The most significant hits for the tumors were the TF PITX1, MITF, AR, and TFAP2C. Previously, we performed a pan-cancer analysis on *TERT* regulation, analyzing a comprehensive dataset of transcription profiles of 19 cancer types using the same computational method [17]. In this pan-cancer analysis, we identified TF as being specific for a single tumor type as well as common TF. Comparing the models of PCa versus all other 18 cancer types led (also) to 17 TF being specific for *TERT* regulation in PCa (from the 17 regulators, AR and E2F2 were also found in several other cancer entities (=common regulators)) (Table S2). We observed a high overlap, i.e., 12 TF, which were found in both analyses (marked with * in Table 1 and in Table S2). We selected these 12 TF serving as a short list of predicted direct regulators of *TERT*. In both studies, PITX1 was the most significant direct regulator of *TERT* for PCa. Notably, PITX1 is not a common *TERT* regulator within the investigated tumor entities. Over all 19 cancer types analyzed, it was a significant *TERT* regulator in only a few other cancers comprising head and neck carcinoma, ovary, and cervical cancer [17]. In summary, we assembled 12 TF predicted to be direct regulators of *TERT* in PCa, and among them, PITX1 was the most prominent.

### 3.2. Identifying a Regulatory Module for TERT Regulation in PCa

TF are highly interacting with other TF or co-factors, enabling a fine-grained homeostasis of gene regulation, and particularly of pace making genes [18,50]. To infer such a regulatory network for *TERT* regulation, we used the 12 identified TF (identified as described in the previous section). To add indirect regulators of *TERT*, which directly regulate these 12 direct *TERT* regulators, we first applied MIPRIP separately for the coding gene of each of the 12 direct regulators and selected all TF predicted by at least 20% of the models (Table 2). This resulted in 72 TF predicted to regulate the 12 direct *TERT* regulators. For TFAP2D, no TF could be predicted as no expression values were available in the transcription profiles. Remarkably, 27 out of these 72 indirect regulators were also potential direct regulators of *TERT* (marked with * in Table 2). PITX1 showed the highest overlap of indirect and direct TF of *TERT* (5 out of 10). Next, we combined the direct and indirect regulators and constructed models following two objectives, i.e., (i) selecting direct *TERT* regulators with which the model fit best the expression profiles of *TERT* across all investigated tumor samples (best fit of MIPRIP), and (ii) obtaining the most densely connected regulatory module (highest modularity) when adding TF from the pre-selected twenty-seven indirect *TERT* regulators. The tradeoff between these two objectives of a good model of direct *TERT* regulators versus a good modularity model was gauged by the weighting factor λ. For low λ values, the MIPRIP objective dominated the models, while high λ values led to a modularity-driven regulator selection resulting in a high number of indirect regulators and an insufficient prediction of *TERT* expression. To obtain the best balance, we compared the number of indirect and direct *TERT* regulators in the models and selected the model with the best balance (λ = 1.188, see Figure 1A). With this selection of the parameter, we observed a good prediction performance of *TERT* expression (Pearson’s correlation was *r* = 0.48 between model and experimentally (RNA-seq) derived expression values of *TERT*). For higher λ values, the performance dropped considerably (*r ≈* 0.25) (Figure 1B). Furthermore, we observed that at least six direct regulators were necessary to obtain a good prediction of *TERT* expression in the MIPRIP models (Figure 1C). Regarding the combinations of six direct regulators used most often over all models (after performing a 10-times three-fold cross-validation across several scales of the model, Figure 1D), led to the six direct regulators BHLHE40, CTCF, IRF1, MITF, PITX1, and TFAP2D (counted in 19% of the models (*n* = 108), *p*-value < 2.2E-16). These direct regulators (Figure 1E, marked in red) were most often found in models with the 14 indirect regulators E2F4, MAZ, POLR2A, POU2F2, SMARCB1, TAF1, REST, PML, SMC3, ZNF263, EP300, YY1, MAFK, and USF1 leading to our final gene regulatory network module for *TERT* in PCa (Figure 1E). Out of these 14 regulators, E2F4, MAZ, POLR2A, POU2F2, SMARCB1, TAF1, and REST (marked in orange in Figure 1E) have been observed to also bind directly to the *TERT* promoter (as listed in the ChIP databases). To elucidate if the rest of the predicted indirect regulators were associated with telomere maintenance, we queried the TelNet database, a manually curated collection of telomere maintenance genes [51]. This query pointed to PML, SMC3, and USF1 (see Discussion).

Our gene regulatory modeling analyses led to a gene regulatory network module for *TERT* regulation comprising 6 direct and 14 indirect regulators of *TERT,* very likely controlling the specific regulation of *TERT* in PCa.

### 3.3. PCa Tissue Cells with A High PITX1 Protein Expression Show Higher Telomere Staining Intensity

**Figure 2:**
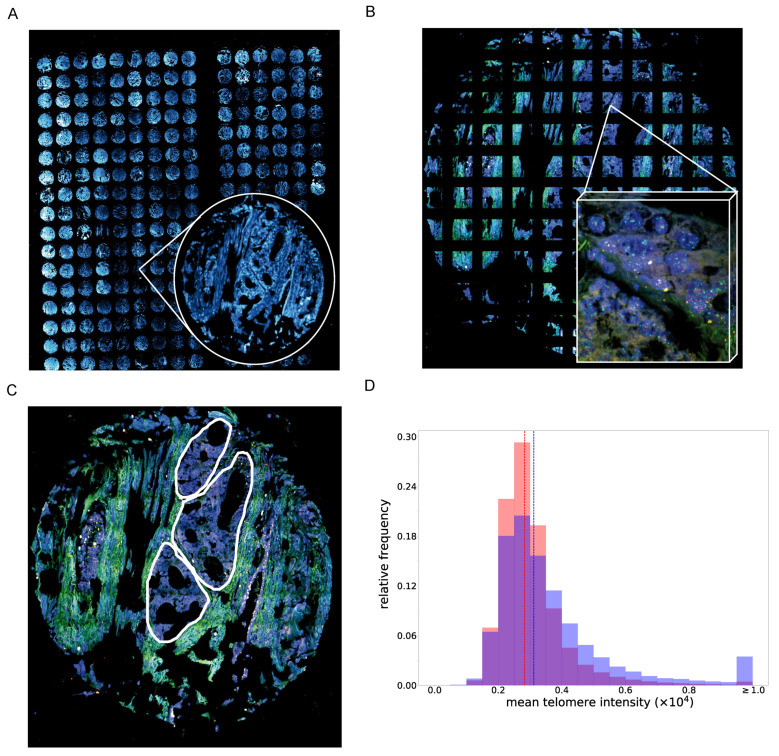
Quantification of telomere length in PCa tissues based on an automated 3D imaging-based workflow. (**A**) Shows an overview of the analyzed TMA. From this TMA, 34 tissue slides of 17 patient samples were imaged representing three different patient sample cohorts: one cohort included all patient samples with a PITX1 status high and high Gleason Score (≥4 + 4), one cohort with PITX1 status low and high Gleason Score (≥4 + 4), and one with PITX1 status low and low Gleason Score (3 + 4). (**B**) For each core, tiled images were acquired and stitched together for the analysis. (**C**) To focus on the tumors, the tumor regions were manually marked by a pathologist. Only tumor regions were considered. (**D**) shows the distribution of mean telomere intensities of cells in samples with high (blue) versus negative (red) PITX1 levels, violet: overlapping events.

Telomere length can be estimated by telomere-staining intensity [52]. We investigated the association between telomere length and PITX1 status. For this, an established automated high-resolution imaging and analysis workflow we developed earlier [52] was applied to prostate tumor tissues on microarrays (tissue microarrays, TMA), focusing on a small, representative subset of patient samples from the immune-histochemical (IHC) analysis described in Methods (Figure 2A). From these TMA, three groups were investigated, i.e., (i) all patient samples with high PITX1 protein expression in the IHC analysis and a high Gleason Score (≥4 + 4, 67,720 telomeres from *n* = 6 patients), a comparable number of samples (ii) with PITX1 status negative and high Gleason Score (≥ 4 + 4, 51,951 telomeres from *n* = 5 patients), and (iii) PITX1 status negative and low Gleason Score (3 + 4, 51,204 telomeres from *n* = 6 patients). For 34 samples (of *n* = 17 patients in duplicate), images were tiled and telomere and PITX1 intensities were considered on tumor regions specified by a pathologist (Figure 2B,C). Indeed, observing over half a million cells (520,847 cells, of these, were 279,410 in tumor regions and 241,437 in non-tumor regions), we found higher averaged telomere-staining intensity indicating longer telomeres in samples with high PITX1 expression compared to samples with PITX1 status negative (*p*-value < 0.001, Mann–Whitney U Test, Figure 2D). This shift was independent of the Gleason Score, i.e., the distributions of PITX1-negative samples with high and low Gleason Score were comparable (Figure S4). To support that tumors with a high PITX1 expression show higher telomere intensities, we investigated publicly available datasets of samples for which gene expression profiles and estimated telomere lengths had been obtained [52,53]. In line with the protein expression, we observed a positive correlation between estimated telomere lengths and *PITX1* gene expression (*r* = 0.33, *p*-value = 0.01 when testing estimated telomere length in PITX1 high versus low gene expression).

In summary, we observed that PITX1 protein and gene expression in primary prostate tumors correlate with longer telomeres. Details about the methods are given in the Supplementary Material Text S1, [54,55,56,57,58].

### 3.4. In Vitro Experiments Showed That PITX1 Binds to the Promoter of TERT and PITX1 Knockdown Reduces TERT Expression

**Figure 3:**
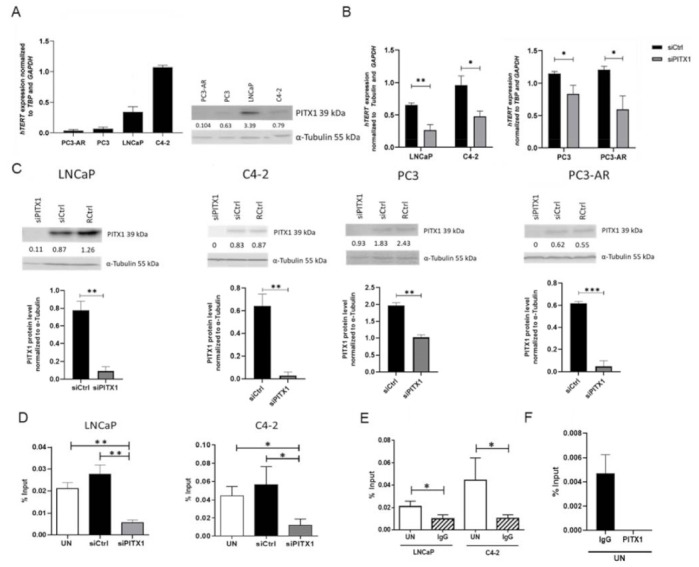
PCa cell lines show divers *TERT* expression levels, decreased *TERT* expression and PITX1 *TERT* promoter binding upon PITX1 knockdown. (**A**) Endogenous *TERT* gene expression was quantified by qPCR in all investigated PCa cell lines, *n* = 3 biological replicates. For each cell line, a representative Western blot is shown. Numbers indicate the PITX1 protein band intensity normalized to α-tubulin. (**B**) *TERT* expression quantified by qPCR in non-targeting pool siRNA-transfected (siCtrl) or PITX1 targeting siRNA pool (siPITX1) transfected PCa cell lines. All cell lines express significantly lower *TERT* levels after PITX1 knockdown, *n* = 3 biological replicates. (**C**) Expression levels of PITX1 after siRNA knockdown (siPITX1) compared to a non-targeting siRNA pool (siCtrl). For each cell line, a representative Western Blot of siRNA knockdown is shown, including reagent control (siCtrl). Numbers indicate the PITX1 protein band intensity normalized to α-tubulin. In addition, a bar graph combining three biological replicates for each cell line is shown. All cell lines reveal a significant knockdown compared to the control. (**D**) ChIP against PITX1 was performed with either untreated (UN), non-targeting pool siRNA-transfected (siCtrl) or PITX1 targeting siRNA pool (siPITX1) transfected in LNCaP (left) and C4–2 (right) cells followed by qPCR. Significantly lower PITX1 binding to the −1.3 kb TERT promoter region compared to UN and siCtrl was obtained in the cells in which PITX1 was knocked down; LNCaP: *n* = 3 biological replicates; C4–2: *n* = 4 values for the statistics obtained from two biological replicates with two technical replicates each. (**E**) Significant binding of the PITX1 antibody in untreated (UN) LNCaP and C4–2 cells in comparison to IgG control (IgG) antibody at the −1.3 kb TERT promoter region, LNCaP: *n* = 3 biological replicates; C4–2: *n* = 4 values for the statistics obtained from two biological replicates with two technical replicates each. (**F**) Negative control of PITX1 hTERT promoter binding (−0.1kb region, no binding site of PITX1) in untreated (UN) LNCaP cells in comparison to IgG control (IgG). For the PITX1 antibody there was no detectable CT value after 40 PCR cycles, *n* = 3 biological replicates, shown are mean and standard deviation (*p* ≤ 0.05 *, *p* ≤ 0.01 ** and *p* ≤ 0.001 ***).

To validate our modeling predictions for PITX1, we selected a collection of four PCa cell lines showing different levels of *TERT* expression (Figure 3A). PC3-AR and PC3 expressed *TERT* rather low compared to LNCaP and C4–2, C4–2 showed the highest *TERT* expression. As hTERT mRNA expression is strictly controlled and closely associated with telomerase activity [53,59,60], we used TERT mRNA levels as a proxy for TERT protein levels and telomerase activity. Like-wise endogenous PITX1 protein levels were investigated, revealing the highest PITX1 expression in LNCaP and lowest in PC3-AR (Figure 3A). Correlating the protein expression of PITX1 and gene expression of *TERT* yielded on average an expected positive correlation (*r* = 0.09), however, not a strong positive correlation. PITX1 protein expression and *TERT* gene expression correlated high for PC3-AR, PC3, and LNCaP cells (*r* = 0.70), however, the overall correlation was lower, as PITX1 protein expression of C4–2 cells was comparably low to their exceptional high *TERT* gene expression. A further investigation of this can be interesting but was beyond the scope of our study. An efficient knockdown of PITX1 in all PCa cell lines showed significant downregulation of *TERT* expression compared to the control (Figure 3B,C). This shows that, as suggested by our modeling approach, PITX1 plays an important role in the expression of *TERT* in PCa cells and that it can act as a positive regulator. Chromatin immunoprecipitation (ChIP) showed that PITX1 directly binds to the *TERT* promoter at the −1.3kb region in the PCa cell lines LNCaP and C4–2 (Figure 3D) since upon knockdown of PITX1, PITX1 bound to the DNA was significantly decreased, validating our predictions. As a negative control and to show specific binding, the −0.1kb *TERT* promoter region was additionally investigated, leading to no detectable qPCR signal besides in the input samples (Figure 3E,F).

In summary, the experimental validations of PITX1 directly regulating *TERT* in PCa cells are in good agreement with our modeling predictions.

### 3.5. The Identified Transcription Factor PITX1 Suits as a Prognostic Marker

**Figure 4:**
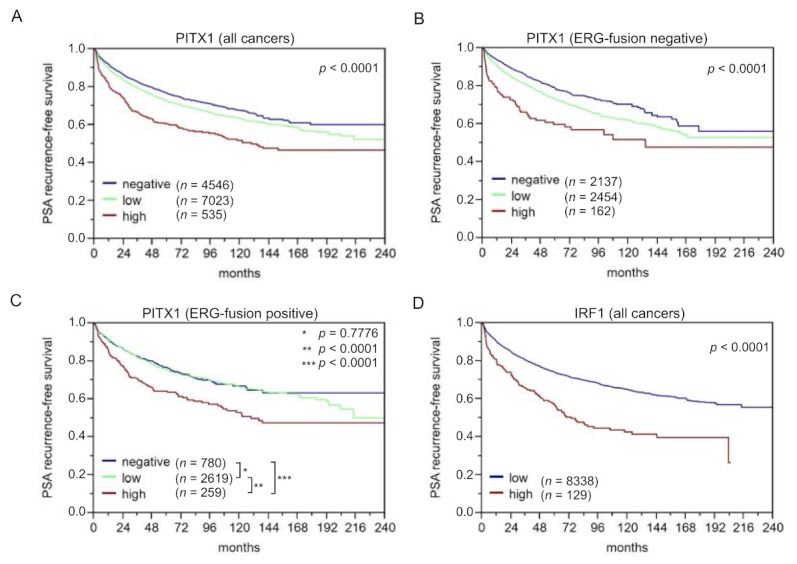
Kaplan–Meier curves of PITX1 high, low, and no protein expression over all patients (**A**) ERG-fusion positive (**B**), and negative subgroups (**C**). (**D**) shows the Kaplan–Meier curve for IRF1 protein expression over all patients. The PSA-recurrence free-survival was used as the primary endpoint.

The six direct *TERT* regulators from the regulatory subnetwork, PITX1, CTCF, IRF1, TFAP2D, MITF, and BHLHE40, were investigated for their prognostic power. For this, TMA of more than 15,000 PCa patients was used for an IHC analysis of these TF. The staining intensities (mostly categorized as negative, low, and high expressed) were correlated with PSA-recurrence-free survival. Additionally, histopathological and molecular variables were studied (e.g., ERG-fusion gene status, *PTEN* deletion). For PITX1, the staining was observed in the nucleus and in the cytoplasm. PITX1 staining was evaluable for 15,011 tumor samples on TMA. Figure S1A–C shows representative images of PITX1 immunostaining in tumor samples with a negative, low, and high PITX1 level. A total of 4% of the tumor samples (*n* = 600) showed a high PITX1 level, 57.7% (*n* = 8,661) were classified as low and 38.3% (*n* = 5749) as negative (Table 3). A Kaplan–Meier analysis revealed that patients with a high PITX1 level had distinctive lower PSA-recurrence free survival compared to patients with a low or negative PITX1 level (*p*-value < 0.0001) (Figure 4A). As the number of patients with high PITX1 levels was low, we obtained a rather low sensitivity but high specificity when using PITX1 level low and negative as a predictor for five years PSA recurrence-free survival (7% sensitivity, 96% specificity). Using only PITX1 level negative as a predictor of PSA recurrence-free survival, sensitivity was 68% and specificity 39%.

Compared to PITX1 negative, low or high PITX1 level (in the following denoted as samples with a positive PITX1 level) was associated with higher tumor aggressiveness including advanced tumor stage (*p*-value < 0.0001), higher Gleason grade, more presence of lymph node metastasis, and more positive surgical margin (*p*-value < 0.0001 each). Furthermore, PITX1 was strongly linked to the presence of ERG fusion: nearly 80% of ERG positive tumor samples (71.6% low and 6.9% high) but only 55% of ERG negative tumor samples (51.5% low and 3.3% high) (*p* < 0.0001; Tables S3 and S4) showed immune-histochemical PITX1 expression. To address the relationship between PITX1 and genomic instability, we compared data on recurrent deletions prevailing in ERG-fusion positive (10q23, PTEN; 3p14, FOXP1) [37,38] or ERG-fusion negative PCa (5q21, CHD1; 6q15, MAP3K7) from a previous analysis of the TMA [39,40]. It showed that PITX1 positivity was strongly linked to all four deletions (*p* < 0.0001 each) in ERG-negative cancers, while this association was lost in ERG-positive cancers, most likely because of the general upregulation of PITX1 in this subset (Figure S2A–C). Additionally, PITX1-positive cancers showed a higher Ki67 index pointing to accelerated cell proliferation, and this was independent of the Gleason grade (Table S5). The patient’s outcome was independent of the ERG-fusion gene status (Figure 4B,C). To determine whether PITX1 can provide an added value to the established prognostic parameters, four different multivariate models were calculated to resemble typical clinical scenarios (Table S6). Scenario 1 utilizes all available parameters after radical prostatectomy (pathological tumor stage, Gleason grade, lymph node, and surgical margin status, as well as pre-operative PSA level) and PITX1 level. Scenario 2 excludes the nodal status, as lymph node dissection is not standardized in surgical PCa therapy. Scenarios 3 and 4 model the pre-operative situation, taking into account that the Gleason grade of the biopsy is often underestimated. Scenario 3 included PITX1 level, clinical tumor stage, pre-operative PSA level, and the “true” Gleason grade (which, however, can only be obtained from the prostatectomy specimens), while in scenario 4, the less reliable Gleason grade obtained from the biopsies were used. In general, the postoperative determination of the Gleason grade is more precise than the pre-operative determination [61]. It turned out that PITX1 expression contributed significantly, particularly in scenario 4, supporting its complementary role to PSA, tumor stage, and Gleason grade from the biopsy, i.e., to variables being available before prostatectomy. The best improvement of AUCROC was for scenario 4 in ERG-negative cancers by 0.9% (Table S6).

In summary, PITX1 expression in tumor samples suits as a biomarker for the prognosis of PCa progression, particularly in combination with the pre-operative variables PSA, Gleason grade from biopsies, and tumor stage.

### 3.6. The Identified Transcription Factors IRF1, CTCF, and TFAP2D Also Suit as Prognostic Markers, Particularly when Combining them with PITX1

Furthermore, we analyzed CTCF, IRF1, and TFAP2D immunohistochemically based on the same patient cohort. For IRF1, only 2% of the patients showed a high IRF1 level, but these were significantly associated with poorer PSA-recurrence-free survival compared to patients with a low IRF1-staining intensity (*p* = 0.0001, Figure 4D). The CTCF and TFAP2D analysis were published by us elsewhere recently. Briefly, high CTCF expression is associated with a poor outcome, in particular, for ERG-fusion negative PCa. In a significant fraction of PCa patients, CTCF showed high expression and was associated with tumor aggressiveness (high Gleason grade, advanced tumor stage, lymph node metastasis, early biochemical recurrence, as well as accelerated cell proliferation) (details are given in [45]). For TFAP2D, we observed that the TFAP2D level was high in about 75% of the patients with poorer clinical course, details see [44].

For the remaining predicted TF, BHLHE40, and MITF, an immune histochemical staining analysis could not be performed since no suitable antibodies were available. In summary, four out of six identified PCa-specific *TERT* regulators from the gene regulatory model suit well as novel prognostic markers for PCa. These four markers can support decision-making. Combining the identified prognostic markers further improved the prediction. A high expression of these four markers showed a strongly decreased PSA-progression free-survival compared to a high expression of only two or less of these markers. For this, specific scores for a negative, weak, moderate or strong staining signal were summed up, and a Kaplan–Meier analysis was performed for differently summed scores (Figure S3). In summary, the modeling analysis identified four new, valuable prognostic markers, and particularly PITX1, for prognosis of PCa progression.

## 4. Discussion

We applied a new network modeling approach to a comprehensive transcription profiling dataset of PCa and identified a gene regulatory network module of direct and indirect regulators (transcription factors) for regulating *TERT*. This module predicted six direct *TERT* regulators, i.e., BHLHE40, CTCF, IRF1, MITF, PITX1, and TFAP2D, and 14 indirect regulators regulating *TERT* expression through the direct *TERT* regulators.

As the most significant direct regulator of *TERT*, we identified PITX1. We experimentally studied the impact of PITX1 on telomere lengths and *TERT* expression. Investigating more than 500,000 cells on tissue slides, we found longer telomere lengths in samples with high compared to low PITX1 protein expression. Chromatin immunoprecipitation showed that PITX1 binds to the *TERT* promoter at the −1.3 kb region in PCa cells to our knowledge for the first time. Furthermore, within a functional assay, we observed *TERT* to be down-regulated when knocking down PITX1. From these observations, we conclude that PITX1 is an activator of *TERT* and hence supports telomerase activity in PCa, suggesting to enable higher proliferation. Indeed, we observed higher Ki-67 values in tumor samples from patients with non-negative PITX1 protein expression, indicating higher proliferation of these tumors. It has been shown that PITX1 regulates *TERT* also in cells of other cancer types. Qi et al. imported human chromosome 5 into the murine melanoma cell line B16F10 using microcell-mediated chromosome transfer [49]. They observed that human PITX1 could directly bind to the *mtert and hTERT* promoter (one binding site at the *mtert*, three at the *hTERT* promoter). Recently, it was reported that interaction of PITX1 and ZCCHC10 contributes to *TERT* regulation in melanoma cells [62], and it was shown that PITX1 binds to the *TERT* promoter in gastric cancer cells [63]. In a functional study, Ohira et al. showed that PITX1 was directly regulated by microRNA-19b (miR-19b), and inhibition of PITX1 expression by miR-19b mimics was associated with increased *hTERT* transcription and proliferation in human kidney cells [64]. Hence, PITX1 was rather described as a suppressor of *TERT* expression and telomerase activity in cells of this other cancer type. In turn, we observed it to act as an activator supporting its oncogenic role in PCa. Originally, PITX1 was described as a developmental morphogenic factor [65]. Its ambivalent role may be linked to its developmental role. This needs further investigation in future studies.

In addition, in clinical oncology, PITX1 has been associated with very controversial roles. Several studies have identified PITX1 as a tumor marker for an unfavorable clinical course. Based on 347 normal lung tissues and 483 tissues of lung adenocarcinomas, Zhang et al. found that the mRNA level of PITX1 was significantly higher in patients with lung adenocarcinoma than in controls, and this association was validated on the protein level performing Western blots for *n* = 12 patients [66]. Similarly, high PITX1 expression was associated with poor prognosis in lung adenocarcinoma [67] and head and neck squamous cell carcinoma [68]. In contrast, PITX1 over-expression was associated with a more favorable outcome of osteosarcoma [69], colorectal [70], gastric cancer [63], and esophageal squamous cell carcinoma [71]. Kolfschoten et al. observed high PITX1 gene expression in normal prostate, muscle, lung, and kidney tissue when comparing 12 different tissues, and, based on a rather small dataset of *n* = 52 samples from prostate tumors, they found PITX1 to be relatively lower expressed in the cancer material compared to normal tissue [72]. Experimentally, they showed that PITX1 suppresses cell growth of human fibroblasts, mediated by downregulation of the RAS pathway through RASAL1. Furthermore, they showed that knockdown of PITX1 in a prostate cell line induced its cell growth. To note, this experiment was conducted in RWPE1 cells, which were immortalized prostate cells, not originating from tumors (HPV induced immortalization and readily transformed by oncogenic K-RAS). Liu and Lobie observed that PITX1 activates p53 in breast cancer cells leading to induced cell cycle arrest and apoptosis [73]. Here, we observed PITX1 protein to be expressed in approximately two-thirds of more than 15,000 PCa samples in contrast to samples from normal prostate tissue, in which it was low or not expressed. This seems in contradiction to the observations by Kolfschoten et al. and needs future investigations. We identified PITX1 as a prognostic marker for PCa. Tissue slides of a comprehensive set of more than 15,000 prostate tumors showed that a low or high level of PITX1 was associated with a poorer prognosis compared to PITX1 negative status. Along with this, for this patient group, we found higher Ki67 level (indicating higher cell proliferation) and higher tumor aggressiveness (advanced tumor stage, higher Gleason Score, more presence of lymph node metastasis, higher levels of positive surgical margin).

We also predicted CTCF, IRF1, TFAP2D, BHLHE40, and MITF as direct regulators of *TERT*. As reported recently, we identified the CCCTC-binding factor (CTCF) as a good prognostic marker specifically for patients with ERG-negative PCa [45]. IHC staining of TFAP2D (details, see [44]) and IRF1 showed that both transcription factors also suited as prognostic markers for PCa. For the predicted direct regulators MITF and BHLHE40, the immunohistochemical staining did not work in our assays. As a future aspect, we suggested the development of new antibodies for these potential biomarkers. Hence, four out of six direct regulators of the identified gene regulatory module suited very well as prognostic markers. Combining them in a linear regression model further improved the predictive power.

The gene regulatory network module consisted of 14 indirect regulators. Seven out of these indirect regulators (E2F4, MAZ, POLR2A, POU2F2, SMARCB1, TAF1, and REST) can potentially bind to the *TERT* promoter and may hence regulate *TERT* also directly, but this needs further experimental investigation. The identified indirect regulators SMC3, PML, and USF1, have been described in the context of telomere maintenance [51]. USF1 is a telomerase activating and repressive factor reported to bind to the human *TERT* promoter regulating its expression [16]. SMC3 has been described to mediate chromosome cohesion, DNA replication, and DNA repair, and it was found at telomeres of telomerase-positive HeLa cells [74,75]. Thus far, PML was described to be rather involved in alternative telomere maintenance mechanisms (ALT). It was observed to localize to telomeres in ALT-positive cells, forming an ALT-associated PML body (APB) [74,76]. PML nuclear bodies were proposed to promote telomeric recombination in ALT cells [77]. We suggest now also a regulatory role of PML for canonical telomere maintenance.

Until now, the PSA level in blood and Gleason grading of tumor samples are the best-established parameters for the diagnosis and prognosis of PCa. Particularly, the postoperative determination of the Gleason grade investigating the dissected tumor sample can be very precise [61]. Recently, we suggested an optimized Gleason grading (quantitative Gleason or IQ Gleason), leading to a more refined estimation of patient prognosis compared to conventional Gleason grading [78]. In turn, pre-operative prediction based on biopsies can be rather error-prone [61], as, by chance, biopsies may not reveal the actual aggressiveness of the tumor when sampled from inappropriate sites. Hence, it is clinically highly relevant to find better means to predict a patient’s clinical course before surgery. The data of this study suggest that PITX1, and probably even superior, the combined analysis of the four identified key transcription factors may substantially improve estimating patient prognosis and can potentially aid in clinical decision making. Our study is based on a large collection of more than 15,000 tumor samples. Still, in the future, a clinical trial is necessary to prospectively prove our biomarkers in the daily clinical routine and particularly assess their power when based on biopsy samples.

Methodologically, we developed a new Mixed Integer Linear Programming based approach to construct a gene regulatory network elucidating the regulation of *TERT* expression. Mixed Integer Linear Programming can be applied to a large range of problems, including time table optimization, solving the traveling salesman problem, Flux Balance Analysis [79], analyses of cell-networks [80,81], classification [82], or inferring gene regulation [24,27]. In a MILP framework, problems can be combined straightforwardly by adding their lists of constraints. As regulators are highly interacting with each other or other co-factors to regulate the expression of a particular gene, we considered not only the regulators directly binding to the promoter of the particular gene but we also integrated TF directly interacting with the ‘direct’ regulators and hence indirectly with the target gene. Using this approach, we identified a highly connected subnetwork, with which we could best predict the gene expression profile of *TERT* in the direct gene regulatory model while maximizing the sum of edge weights between the connected nodes of the indirect network. These two objectives were balanced, leading to high connectivity in the regulatory module and accurate *TERT* expression prediction. This approach is generic and can be applied to any other disease or investigated biological condition for which such gene regulatory modules of direct and indirect regulators are of interest, as long as sufficient transcription profiles are available.

As negative controls, we used expression data of samples annotated as normal tissues. Interrogating the original study [3], these samples are described as adjacent to the prostate tumor samples. Using patient-matched normal controls when compared with tumors is generally an advantage. However, this may also have the limitation of the presence of some tumor cell infiltration or infiltration of other cells of the tumor microenvironment and is a likely explanation for the detectable levels of *TERT* expression in these samples. In line, our modeling analysis also predicted *TERT* regulators for these samples, which may be reasoned by such an infiltration. We see the clinical application of our results primarily in PITX1 serving as a new biomarker for prostate cancer rather than as a therapeutic target. In initial cell assays, we could not see a significant reduction in the growth rate when knocking down PITX1. For this, we observed the optical density until five days after knockdown. As the effects of reduced telomerase activity on growth may be seen only after many cell divisions (when cells obtain senescent due to too shortened telomeres), future studies may be needed to shape long-time effects.

## 5. Conclusions

In summary, it is known that *TERT* does not suit as a prognostic marker for PCa as it is very low expressed. Still, *TERT* is essential for PCa cells. It is needed for telomere maintenance, as no alternative telomere maintenance (ALT) mechanism has been observed. We investigated if, instead of *TERT*, we find regulators suiting as prognostic markers. Constructing and applying a new gene regulatory network module considering directly acting TF and TF indirectly regulating the expression of *TERT* by interacting with directly regulating TF, we predicted six TF directly regulating *TERT*. Investigating stained tumor samples, four out of these six TF suit as good prognostic markers for PCa progression, and particularly when the information of their protein expression on these tumor samples is combined. Our most prominent hit, PITX1, was experimentally validated acting as an activating TF of *TERT* expression in PCa cells. For the future, a clinical trial is necessary to prove PITX1 as a new biomarker for the daily clinical routine, particularly when using pre-operative biopsies. In addition, the mechanistic link of PITX1 mediated *TERT* regulation and survival needs more elaborative future investigations.

## Figures and Tables

**Table 1:** Significant *TERT* regulators of PCa compared to normal prostate tissue and vice versa.

Regulators Tumor	Frequency Tumor (*n* = 300 Models)	Frequency Normal (*n* = 300 Models)	*p*-Value **
PITX1 *	186 (62%)	35 (12%)	1.56 × 10^−37^
MITF *	119 (40%)	28 (9%)	5.97 × 10^−17^
AR *	92 (31%)	21 (7%)	1.26 × 10^−12^
TFAP2C *	72 (24%)	11 (4%)	1.67 × 10^−12^
E2F2 *	92 (31%)	24 (8%)	1.31 × 10^−11^
NR2F2 *	97 (32%)	27 (9%)	1.31 × 10^−11^
SMARCB1	88 (29%)	24 (8%)	1.15 × 10^−10^
CEBPA *	65 (22%)	20 (7%)	6.08 × 10^−7^
BHLHE40 *	53 (18%)	16 (5%)	8.26 × 10^−6^
CTCF *	48 (16%)	15 (5%)	4.13 × 10^−5^
ETS1 *	63 (21%)	26 (9%)	7.43 × 10^−5^
MXI1	27 (9%)	5 (2%)	1.75 × 10^−4^
POLR2A	34 (11%)	9 (3%)	2.23 × 10^−4^
RAD21	32 (11%)	11 (4%)	2.37 × 10^−3^
IRF1 *	31 (10%)	12 (4%)	6.38 × 10^−3^
TFAP2D *	34 (11%)	18 (6%)	3.91 × 10^−2^
MAX	36 (12%)	20 (7%)	4.62 × 10^−2^

* marked TF were predicted as *TERT* regulators specifically for prostate cancer in a previous study of us [17]. ** adjusted for multiple testing correction (Benjamini-Hochberg).

**Table 2:** MIPRIP analysis of the 12 identified prostate-specific *TERT* regulators.

*TERT* Regulator	Regulators Used in At Least 20% of the Models	Number of Direct Regulators	Number of *TERT* Regulators
PITX1	SMARCC1, TAF1 *, HEY1 *, POLR2A *, FOXO1, HNF4A, ESR1 *, RBBP5, SMAD1, SMARCB1 *	10	5
AR	MAFF, MAFK, ZBTB17, CREB3, GATA2, TCF4, CTCF *, EGR1 *	8	2
MITF	MXI1 *, ZNF263, SMC3, TAL1 *, MYC *, EP300, MAX *	7	4
CTCF	MAX *, PRDM16, YY1, RBBP5, REST *, POU2F2 *, FOXP2, EP300	8	3
BHLHE40	ARNTL, HIF1A *, SIN3AK20 *, EGR1 *, NCOR1, AR *, CEBPB, GABPA, ZNF143	9	4
ETS1	ETV2, PAX5 *, FOS, CEBPB, USF1, FOXA1, TCF7L2, IRF4, GATA2	9	1
CEBPA	SP1, CLOCK, IKZF1 *, MYC *, NCOR1, FOXP2, JUN, SREBF1, MAZ *	9	3
E2F2	E2F4 *, PML, E2F7, MAFK, ELF1, HEY1 *, EBF1, E2F6 *, MAFF, TCF12 *	10	4
NR2F2	MXI1 *, TP53 *, USF1, E2F4 *, SF1, FOXP2, SIN3AK20 *, ZNF263	8	4
IRF1	NFKB.P50.P65 *, IRF2, SPI1, EGR1 *, MYB *	5	3
TFAP2C	TP63, MAX *, RAD21 *, RBPJ, SP1, POU5F1, ZFP36L1, MTA1, E2F1 *, EZH2, SETDB1	11	3

* TF binding to the *TERT* promoter (potential direct regulators of *TERT*).

**Table 3:** Association of PITX1 expression in tumor tissues and PCa characteristics.

	PITX1				
Parameter	*n* Evaluable	Negative (%)	Low (%)	High (%)	*p* Value
All cancers	15,011	38.3	57.7	4.0	
Tumor stage					<0.0001
pT2	9555	41.5	55.5	3.0	
pT3a	3366	34.6	60.4	5.0	
pT3b-pT4	2030	30.0	63.0	7.0	
Gleason grade					<0.0001
≤3 + 3	2794	41.8	55.3	2.8	
3 + 4	7971	40.2	56.5	3.3	
3 + 4 Tert.5	720	38.9	57.6	3.5	
4 + 3	1479	30.6	62.8	6.6	
4 + 3 Tert.5	1056	31.3	63.5	5.2	
≥4 + 4	867	28.7	61.5	9.8	
Lymph node metastasis					<0.0001
N0	9067	37.7	58.0	4.3	
N+	1121	30.2	63.2	6.6	
Surgical margin					<0.0001
negative	11,973	39.2	57.1	3.7	
positive	2985	35.1	59.8	5.1	

## Data Availability

All expression data and data from chromatin immune precipitation used for model building is publically available as described in Methods. The basic gene regulatory network model bases on MIPRIP 2.0, which is implemented as a software package in R [17,24]. It is freely available on github (https://github.com/KoenigLabNM/MIPRIP, accessed on 10 January 2022). MIPRIP 2.0 is platform-independent and runs on R version 3.5.1 together with Gurobi version 7.0.1 and the CRAN R package slam. Extension of the model by the modularity approach was performed using custom scripts (as described in Methods), which do not need a specific software package implementation. All experimental cell assay data generated or analyzed during this study are included in this article and its supplementary information.

## Supplementary Materials

The following are available online at https://www.mdpi.com/article/10.3390/cancers14051267/s1, Figure S1. Representative images of PITX1 staining of PCa, Figure S2. Association between PITX1 and PTEN, 6q15, 5q21, and 3p13 deletions, Figure S3. Kaplan–Meier curve of the combination of all four markers, Figure S4. Quantification of telomere length in the three different groups based on an automated 3D imaging-based workflow. Table S1. Significant TERT regulators of normal prostate tissue (control) versus PCa tissue to identify tumor-specific regulators, Table S2. Significant TERT regulators of PCa compared to 18 other cancer types, Table S3. Association between PITX1 immunostaining results and PCa phenotype in ERG–fusion negative tumors, Table S4. Association between PITX1 immunostaining results and PCa phenotype in ERG–fusion positive tumors, Table S5. Association between PITX1 expression and Ki67-labeling index in (a) all, (b) Gleason grade ≤ 3 + 3, (c) Gleason grade 3 + 4, (d) Gleason grade 4 + 3, (e) Gleason grade ≥ 4 + 4, (f) PTEN norm, (g) PTEN del PCa, Table S6. Multivariate analysis including PITX1 expression in (a) all cancers, (b) ERG-negative and (c) ERG-positive cancers, Text S1. Associating telomere length to PITX1 expression employing microarrays of tissue slides of PCa.
