# Supplementary material for: PITX1 Is a Regulator of TERT Expression in Prostate Cancer with Prognostic Power
Source: Cancers (Basel). 2022 Mar 1;14(5):1267. doi: 10.3390/cancers14051267 (PMC8909694; doi:10.3390/cancers14051267)
Supplement: Supplementary file 1 [file cancers-14-01267-s001.zip › cancers-1468357-supplementary.pdf]

PITX1 is a regulator of TERT expression in prostate cancer with prognostic power

Alexandra M. Poos [1,2], Cornelia Schroeder [3], Neeraja Jaishankar [4,5], Daniela Röll [4,5], Marcus Oswald [4], Jan Meiners [3], Delia M. Braun [2], Caroline Knotz [2], Lukas Frank [2], Manuel Gunkel [6], Roman Spilger [7], Thomas Wollmann [7], Adam Polonski [3], Georgia Makrypidi-Fraune [3], Christoph Fraune [3], Markus Graefen [8], Inn Chung [2], Alexander Stenzel [5], Holger Erfle [6], Karl Rohr [7], Aria Baniahmad [5], Guido Sauter [3], Karsten Rippe [2], Ronald Simon [3] and Rainer Koenig [1, 4]

[1] Integrated Research and Treatment Center, Center for Sepsis Control and Care (CSCC), Jena University Hospital, Am Klinikum 1, 07747 Jena, Germany; a.poos@dkfz-heidelberg.de

[2] Division of Chromatin Networks, German Cancer Research Center (DKFZ) and BioQuant Center, Im Neuenheimer Feld 280, 69120 Heidelberg, Germany; d.braun@dkfz-heidelberg.de (D.M.B.); caroline.knotz@dkfz-heidelberg.de (C.B.); l.frank@dkfz-heidelberg.de (L.F.); inn.chung@med.uni-heidelberg.de (I.C.); karsten.rippe@dkfz-heidelberg.de (K.R.)

[3] Department of Pathology, University Medical Center Hamburg-Eppendorf, Martinistraße 52, 20251 Hamburg, Germany; cor.schroeder@uke.de (C.S.); j.meiners@uke.de (J.M.); a.polonski@uke.de (A.P.); g.makrypidi@uke.de (G.M.-F.); c.fraune@uke.de (C.F.); g.sauter@uke.d (G.S.); r.simon@uke.de (R.S.)

[4] Institute for Infectious Diseases and Infection Control (IIMK), Jena University Hospital, Am Klinikum 1, 07747 Jena, Germany; neeraja.jaishankar@uni-jena.de (N.J.); daniela.roell@med.uni-jena.de (D.R.); marcus.oswald@web.de (M.O.)

[5] Institute of Human Genetics, Jena University Hospital, Am Klinikum 1, 07747 Jena, Germany; alexander.stenzel@med.uni-jena.de (A.S.); aria.baniahmad@med.uni-jena.de (A.B.)

[6] VIROQUANT CellNetworks RNAi Screening Facility and Research Group High-Content Analysis of the Cell (HiCell), BioQuant Center, Heidelberg University, Im Neuenheimer Feld 267, 69120 Heidelberg, Germany; manuel.gunkel@bioquant.uni-heidelberg.de (M.G.); holger.erfle@bioquant.uni-heidelberg.de (H.E.)

[7] Biomedical Computer Vision Group, BioQuant Center and IPMB, Heidelberg University and German Cancer Research Center (DKFZ), Im Neuenheimer Feld 267, 69120 Heidelberg, Germany; roman.spilger@bioquant.uni-heidelberg.de (R.S.); thomas.wollmann@bioquant.uni-heidelberg.de (T.W.);
k.rohr@dkfz-heidelberg.de (K.R.)

[8] Martini-Clinic, Prostate Cancer Center, University Medical Center Hamburg-Eppendorf, 20251 Hamburg, Germany; graefen@uke.de

Supplementary material

Text S1. Associating telomere length to PITX1 expression employing microarrays of tissue slides of PCa

To determine whether the telomere length of tumors with different PITX1 levels differs, a tissue microarray (TMA) including a subset of the analyzed IHC patient cohort (246 cores, 2 cores per patient) was used. This subset included 8 patient samples with high PITX1 level, 61 patient samples with low PITX1 level, and 48 patient samples with no PITX1 (negative) based on the IHC analysis described above. The staining of telomeres and centromeres was performed as described previously (Gunkel et al, 2017). The tissue microarray was deparaffinized by incubating the samples three times in xylene for 5 min. For rehydration, the sample was incubated in a reverse ethanol row (2x in 100%, 2x in 95% and 2x in 70% ethanol for 3 min each) and subsequently rinsed in deionized water for 3 min and in 1% Tween20 for 1 min. For antigen retrieval, slides were boiled in 10 mM sodium citrate (in H_20, pH 6) for 9 min. After 20 min cooling at room temperature the slide was briefly washed with deionized water for 1 min. For dehydration, the TMA was immersed twice in 70%, 85% and 100% ethanol for 3 min each and air dried for a few minutes. Denaturation and FISH probe hybridization was performed as follows: PNA hybridization buffer (70% formamide, 10 mM Tris HCl, pH 7.5, 0.1lg/ml salmon sperm DNA) containing 0.1 M of a Cy3-labeled telomere probe (CCCTAA)3(TelC-Cy3, Panagene) and a final concentration of 5 µg/mL of a FAM-labeled PNA probe (ATTCGTTGGAAACGGGA) that is directed against the CENP-B binding site in the centromeric alpha satellite DNA (CENP-B-FAM, PNA Bio) was added to the slide. The TMA was heated to 84°C for 5 min for denaturation and hybridization took place o.n. at 30 °C in a wet chamber. Next, the TMA was washed twice for 15 min with gentle agitation in PNA washed buffer (70% formamide, 10 mM Tris-HCl,pH 7.4), 1 min in 2x SSC, 5 min in 0.1x SSC at 55 °C, 2x 5 min in 0.05% Tween-20 (in 2x SSC) and three times in PBST (0.1% Tween20 in 1xPBS) for 5 min. For immunolabeling of PITX1, an anti-PITX1 antibody (ab70273, Lot: 6R2538F4-13, Abcam, 1:200) was used. First, the TMA was blocked with 10% goat serum for 1 hour at room temperature in a wet chamber and subsequent to a brief washing step with PBST, incubated with the primary antibody (1:250 dilution) for 1 hour at room temperature followed by a second washing step with PBST and incubation with an ATTO633-labeled secondary antibody (1:300) for 1 hour. After re-washing three times in PBS, salts were washed out with deionized water and the TMA was mounted in Prolong gold antifade including DAPI.

From this TMA, 34 cores (17 patient samples, 2 TMA cores each) were imaged with an Andor Dragonfly Spinning Disc Microscope. Patient samples were selected based on the PITX1 protein level (high or negative) and the Gleason score. From 17 patient samples, 6 had a high PITX1 level based on the IHC staining and a high Gleason Score (>= 4+4). The other 11 patient samples were negative for PITX1 in the IHC staining and represent the Gleason groups >= 4+4 (5 patient samples) and 3+4 (6 patient samples). For each core, a tiled image was acquired

covering the full area of the core. Most cores could be covered with a tiling of 11 x 11 at 12.5% overlap for stitching, in some cases up to 13 tiles in one direction were necessary for a complete coverage. Each tile had a dimension of 68.2 µm x 68.2 µm x 10 µm. For four color channels, stacks were acquired with an Andor Ixon EM-CCD (1024 x 1024 pixel) at each tile position with excitations of 640 nm, 561 nm, 488 nm, and 405 nm, respectively, with a stack spacing of 250 nm, resulting in 164 images for each tile equivalent to 345 MB of image data per stack. For a single core, at least 19.481 images equivalent to 40 GB of image data was acquired. For all 34 cores, 832,301 images were acquired in total, yielding 2.5 TB of image data. With Macros written in ImageJ, each image stack was projected to a single plane (maximum projection) and stitched in order to obtain a complete overview image of each core (Figure S4B). In the stitched images the tumor region was marked by a pathologist (Ronald Simon) to distinguish between tumor and normal tissue (Figure S4C). These marked regions were subsequently transferred to the original tiles by Macros written in ImageJ and workflows composed using KNIME (www.knime.org). For the 3D images within each core, 2D maximum intensity projection (MIP) was applied. To distinguish between tumor and normal tissue, the tumor regions were manually marked by a pathologist in the MIP images. These annotated tumor regions were subsequently mapped back to 3D. For each tumor region, slice-wise segmentation of cell nuclei was performed using ASPP-Net (Wollmann, 2018). Afterwards, small objects were removed and small holes were filled using morphological operations. The ASPP-Net was pre-trained using images from Ulman *et al*. (Ulman et al, 2017) and fine-tuned on the target dataset, employing 50 manually annotated images. Training was performed with progressive resizing from one quarter resolution to the original resolution of the images to reduce the computation time. Telomere spots within the segmented cell nuclei were quantified using a 3D model-based approach (Worz et al, 2010). Candidate spots were detected employing an anisotropic 3D Laplacian of Gaussian filter ($\sigma_{x,y}$ = 1.5 voxels, σ_z = 1.0 voxels). For each detected candidate spot, 3D least-squares model fitting with an anisotropic 3D Gaussian intensity model representing the intensity profile of telomere spots was performed. Based on the fitting results, the mean intensity of telomere spots was determined.

For gene expression analysis with regard to telomere length, published data on estimated telomere length information (Barthel et al, 2017) generated by TelSeq (Ding et al, 2014) based on whole genome sequencing data of the TCGA PCa cohort was used. The telomere length was correlated with the *PITX1* gene expression from the TCGA PCa RNA-seq dataset used in our modelling approach. Samples with a telomere length < 0.1 kb were excluded.

Table S1. Significant *TERT* regulators of normal prostate tissue (control) *versus* PCa tissue to identify tumor specific regulators

Regulators normal	Frequency normal (n=300 models)	Frequency tumor (n=300 models)	p-value**
TAF9	53	3	1.60E-12
AP-2	42	1	2.15E-11
ETS2	31	0	2.81E-09
HIF1A	31	0	2.81E-09
E2F5	29	0	1.06E-08
HNRNPK	33	1	1.06E-08
EPAS1	27	0	4.33E-08
TP73	24	0	3.69E-07
CTCFL	33	3	4.91E-07
TFAP2B	30	2	5.57E-07
SMAD3	21	0	2.62E-06
MXD1	27	2	3.59E-06
MYCN	29	3	5.13E-06
ESR1	26	2	6.36E-06
NFAT5	27	3	1.62E-05
RUNX2	20	1	4.50E-05
RELA	25	3	5.07E-05
TP53	27	4	5.80E-05
SP3	24	3	8.50E-05
TAL1	29	6	1.75E-04
EGR1	17	1	2.60E-04
E2F4	29	7	4.41E-04
HMGA2	28	7	7.23E-04
NFKB1-RELA	20	3	7.79E-04
HIF1 complex	21	4	1.41E-03
GRHL2	14	1	1.60E-03
ZBTB48	28	8	1.60E-03
NFX1	31	11	3.62E-03
MZF1	14	2	6.35E-03
PAX8	14	2	6.35E-03
NFATC2	16	3	6.38E-03
GLI1	11	1	9.37E-03
E2F6	31	13	1.12E-02
HEY1	24	9	1.70E-02
TCF7	12	2	1.79E-02
IKZF1	20	7	2.44E-02
ESR2	15	4	2.51E-02
NFKB	9	1	2.90E-02
JUND	28	13	3.12E-02
WT1	10	2	4.83E-02

** Adjusted for multiple testing correction (Benjamini-Hochberg)

Table S2. Significant *TERT* regulators of PCa compared to 18 other cancer types (based on the pan-cancer analysis from (Poos et al, 2019))

TF	p-value**
**PITX1***	2.79E-21
**ETS1***	3.04E-19
**MITF***	2.56E-17
**NR2F2***	8.28E-16
**IRF1***	3.38E-13
**TFAP2D***	4.24E-10
**CEBPA***	2.09E-08
**E2F2***	1.02E-07
**BHLHE40***	5.67E-06
**KLF2**	7.09E-04
**TFAP2C***	2.35E-03
**AR***	5.22E-03
**ZBTB48**	8.13E-03
**NFKB1-RELA complex**	1.62E-02
**CTCF***	2.48E-02
**MEN1**	3.37E-02
**TFAP2A**	4.75E-02

* Overlap to the comparison of prostate cancer *versus* normal prostate tissue
** Adjusted for multiple testing (Benjamini-Hochberg)

Table S3. Association between PITX1 immunostaining results and PCa phenotype in ERG–fusion negative tumors

		PITX1			
**Parameter**	**n evaluable**	**negative (%)**	**low (%)**	**high (%)**	**p value**
**All cancers**	5,186	45.2	51.5	3.3	
**Tumor stage**					
pT2	3,483	48.3	49.0	2.7	<0.0001
pT3a	1,036	43.0	53.7	3.4	
pT3b-pT4	654	32.7	60.4	6.9	
**Gleason grade**					
≤3+3	1,012	51.0	46.7	2.3	<0.0001
3+4	2,782	48.0	49.6	2.4	
3+4 Tert.5	238	45.8	50.8	3.4	
4+3	538	34.8	59.7	5.6	
4+3 Tert.5	316	36.4	58.5	5.1	
≥4+4	296	28.0	62.5	9.5	
**Lymph node metastasis**					
N0	3,011	42.9	53.4	3.7	0.0008
N+	296	33.1	60.1	6.8	
**Surgical margin**					
negative	4,149	45.8	51.0	3.1	0.0915
positive	1,026	42.9	52.9	4.2	

Table S4. Association between PITX1 immunostaining results and PCa phenotype in ERG–fusion positive tumors

		PITX1			
**Parameter**	**n evaluable**	**negative (%)**	**low (%)**	**high (%)**	**p value**
**All cancers**	3,993	21.5	71.6	6.9	
**Tumor stage**					
pT2	2,371	22.4	72.2	5.4	0.0002
pT3a	1,064	20.3	71.5	8.2	
pT3b-pT4	542	20.1	69.4	10.5	
**Gleason grade**					
≤3+3	818	22.6	72.5	4.9	0.0004
3+4	2,279	22.4	71.4	6.2	
3+4 Tert.5	126	19.0	70.6	10.3	
4+3	396	18.2	73.0	8.8	
4+3 Tert.5	215	17.2	73.5	9.3	
≥4+4	156	20.5	64.1	15.4	
**Lymph node metastasis**					
N0	2,285	20.7	71.6	7.7	0.0658
N+	255	20.0	67.8	12.2	
**Surgical margin**					
negative	3,131	22.4	71.3	6.4	0.0084
positive	845	18.6	72.8	8.6	

Table S5. Association between PITX1 expression and Ki67-labeling index in a) all, b) Gleason grade ≤3+3, c) Gleason grade 3+4, d) Gleason grade 4+3, e) Gleason grade ≥4+4, f) PTEN norm, g) PTEN del PCa

ki67		n=	Ki67 Li (mean)	Std.deviation
all p<0.0001	negative	2,158	2.09	0.06
	low	3,676	3.13	0.04
	high	307	3.65	0.15
pGleason ≤3+3 p<0.0001	negative	510	1.71	0.09
	low	751	2.47	0.08
	high	46	2.76	0.30
pGleason 3+4 p<0.0001	negative	1,217	1.99	0.07
	low	2,054	3.00	0.05
	high	155	3.19	0.19
pGleason 3+4 Tert.5 p=0.0017	negative	102	2.53	0.26
	low	141	3.72	0.22
	high	12	3.92	0.76
pGleason 4+3 p=0.0099	negative	170	2.73	0.26
	low	376	3.63	0.17
	high	37	3.92	0.55
pGleason 4+3 Tert. 5 p=0.0268	negative	94	2.98	0.38
	low	193	4.09	0.27
	high	23	4.78	0.78
pGleason ≥4+4 p=0.0147	negative	65	3.28	0.59
	low	157	5.07	0.38
	high	34	5.82	0.82

Table S6. Multivariate analysis including PITX1 expression in all cancers, *ERG*-negative and *ERG*-positive cancers

Tumor subset	Scenario	n analyzable	P-value								ROC-Analysis		
			Preoperative PSA-Level	pT Stage	cT Stage	Gleason grade prostatectomy	Gleason grade biopsy	pN Stage	R Stage	PITX1-Expression	Area under the curve (AUC)		
											Excluding PITX1-Expression	Including PITX1-Expression	Difference
All cancers	1	7.829	<0.0001	<0.0001	-	<0.0001	-	<0.0001	<0.0001	0,0177	0.77899	0.78098	0.00199
	2	11.957	<0.0001	<0.0001	-	<0.0001	-	-	<0.0001	0,0039	0.78816	0.79044	0.00228
	3	11.760	<0.0001	-	<0.0001	<0.0001	-	-	-	0,0045	0.75466	0.75791	0.00325
	4	10.036	<0.0001	-	<0.0001	-	<0.0001	-	-	<0.0001	0.7341	0.73582	0.00172
ERG-negative cancers	1	3.038	0,0011	<0.0001	-	<0.0001	-	0,0002	0,0445	0,02	0.76569	0.76988	0.00419
	2	4.739	<0.0001	<0.0001	-	<0.0001	-	-	<0.0001	0,0115	0.77361	0.77792	0.00431
	3	4.695	<0.0001	-	<0.0001	<0.0001	-	-	-	0,012	0.74006	0.74663	0.00657
	4	4.624	<0.0001	-	0,0001	-	<0.0001	-	-	<0.0001	0.71941	0.72879	0.00938
ERG-positive cancers	1	2.325	0,0027	<0.0001	-	<0.0001	-	0,0813	<0.0001	0.1942	0.79156	0.79343	0.00187
	2	3.644	<0.0001	<0.0001	-	<0.0001	-	-	<0.0001	0.1432	0.79952	0.80201	0.00249
	3	3.578	<0.0001	-	<0.0001	<0.0001	-	-	-	0.2372	0.76435	0.76766	0.00331
	4	3.519	<0.0001	-	<0.0001	-	<0.0001	-	-	0.0125	0.76591	0.77061	0.00470

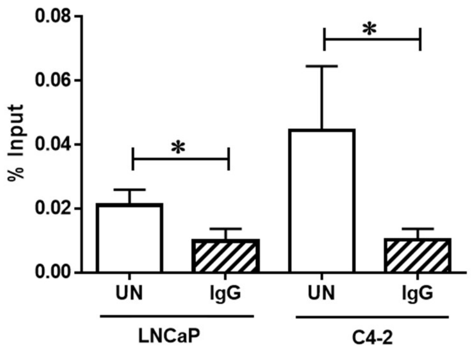

Figure S1. Significant binding of PITX1 antibody in untreated (UN) LNCaP and C4-2 cells in comparison to IgG control (IgG). LNCaP: n=3 biological replicates; C4-2: n=4 technical replicates obtained from 2 biological replicates.

A B C

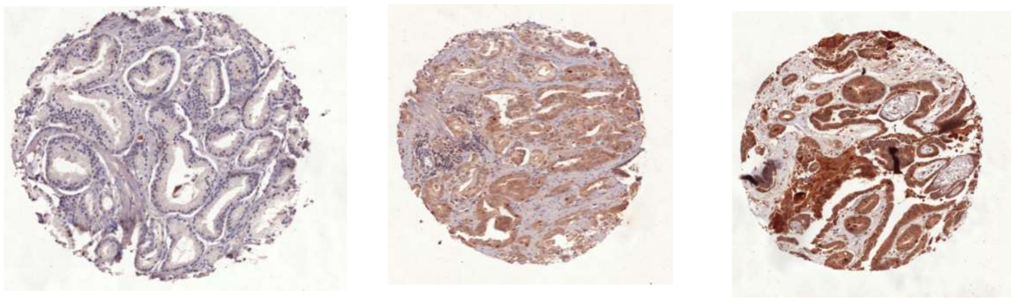

Figure S2. Representative images of PITX1 staining of PCa with A) negative, B) low and C) high expression of PITX1.

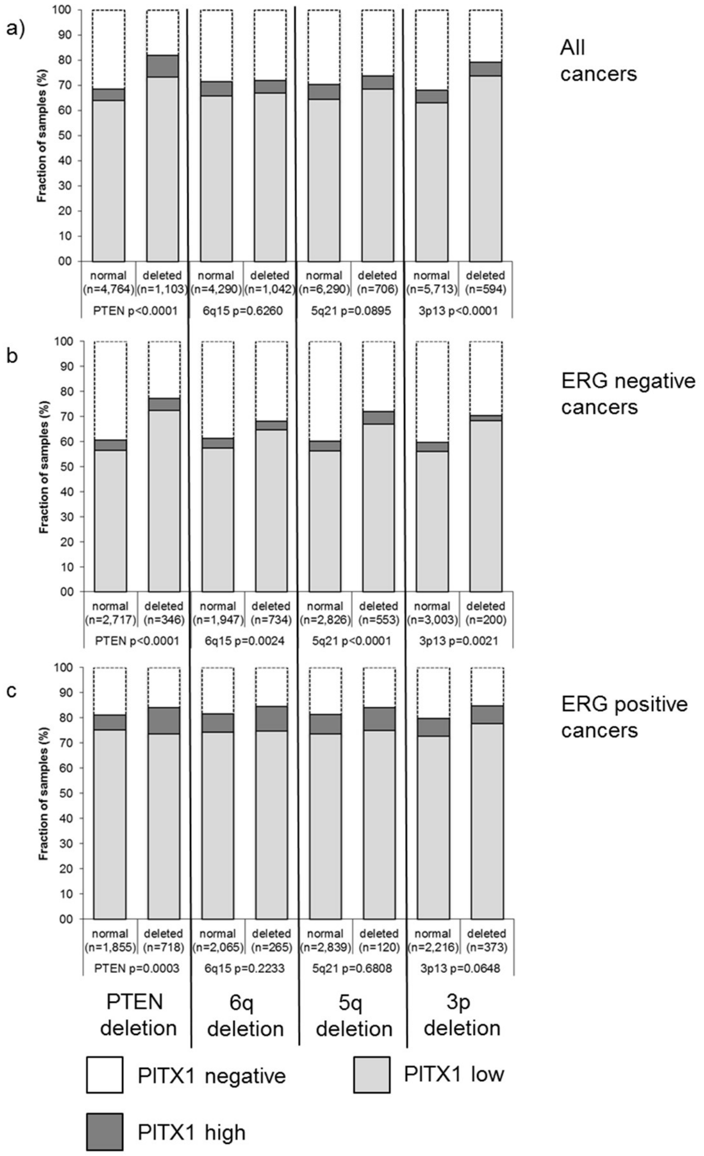

Figure S3. Association between PITX1 and PTEN, 6q15, 5q21 and 3p13 deletions

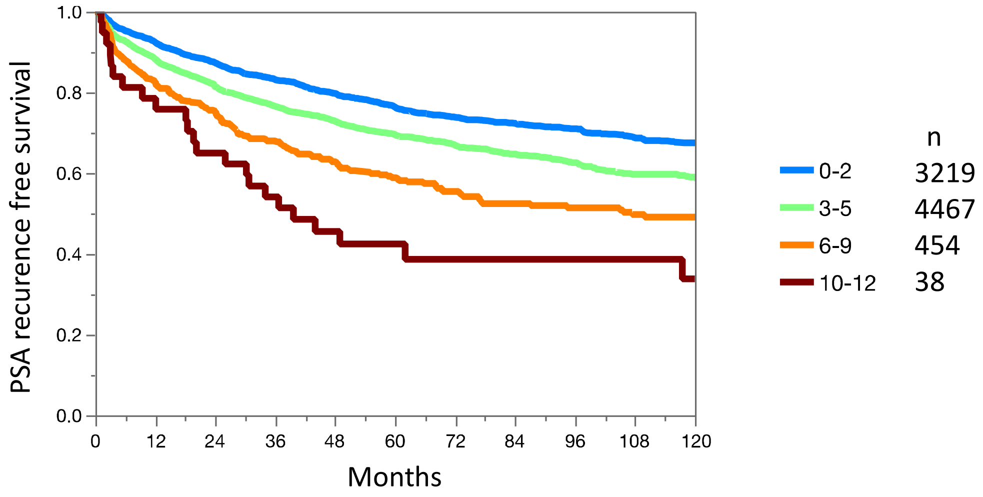

Figure S4. Kaplan Meier curve of the combination of all four markers using the optimized sum of scores for PITX1 (scores 0, 1, 1, 3 for negative, weak, moderate, strong, respectively), CTCF (scores 0, 2, 2, 3 for negative, weak, moderate, strong, respectively), IRF1 (scores 0, 0,0,4 for negative, weak, moderate, strong, respectively) and TFAP2D (scores 0, 1, 2, 2 for negative, weak, moderate, strong, respectively).

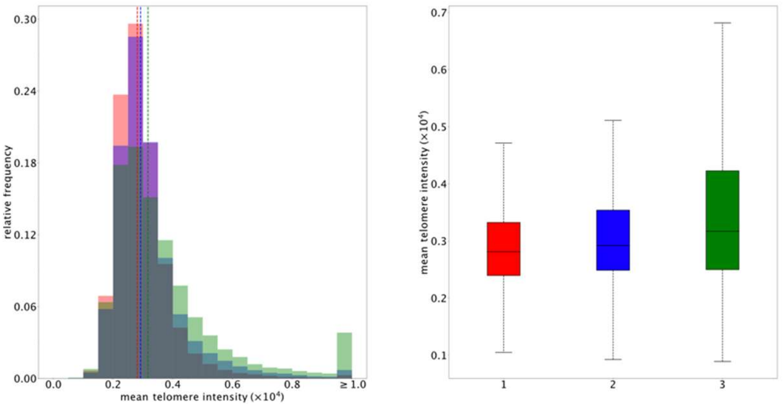

Figure S5. Quantification of telomere length in the three different groups based on an automated 3D imaging-based workflow. (1) PITX1 negative samples with high Gleason score ((≥4+4, red), (2) PITX1 negative samples with low Gleason score ((≥3+4, blue) and (3) PITX1 high samples with high Gleason score ((≥4+4, green). The histogram (left panel) shows the distribution of mean telomere intensities of cells and the right panel the according boxplots.

References

Barthel FP, Wei W, Tang M, Martinez-Ledesma E, Hu X, Amin SB, Akdemir KC, Seth S, Song X, Wang Q, Lichtenberg T, Hu J, Zhang J, Zheng S, Verhaak RG (2017) Systematic analysis of telomere length and somatic alterations in 31 cancer types. *Nat Genet* **49:** 349-357

Ding Z, Mangino M, Aviv A, Spector T, Durbin R, Consortium UK (2014) Estimating telomere length from whole genome sequence data. *Nucleic Acids Res* **42:** e75

Gunkel M, Chung I, Worz S, Deeg KI, Simon R, Sauter G, Jones DTW, Korshunov A, Rohr K, Erfle H, Rippe K (2017) Quantification of telomere features in tumor tissue sections by an automated 3D imaging-based workflow. *Methods* **114:** 60-73

Poos AM, Kordass T, Kolte A, Ast V, Oswald M, Rippe K, Konig R (2019) Modelling TERT regulation across 19 different cancer types based on the MIPRIP 2.0 gene regulatory network approach. *BMC Bioinformatics* **20:** 737

Ulman V, Maska M, Magnusson KEG, Ronneberger O, Haubold C, Harder N, Matula P, Matula P, Svoboda D, Radojevic M, Smal I, Rohr K, Jalden J, Blau HM, Dzyubachyk O, Lelieveldt B, Xiao P, Li Y, Cho SY, Dufour AC et al (2017) An objective comparison of cell-tracking algorithms. *Nat Methods* **14:** 1141-1152

Wollmann TI, J.; Gunkel, M.; Chung, I.; Erfle, H.; Rippe, K.; Rohr, K. (2018) Multi-channel Deep Transfer Learning for Nuclei Segmentation in Glioblastoma Cell Tissue Images. *Proc Bildverarbeitung für die Medizin, Informatik aktuell, Springer Berlin Heidelberg*: 316-321

Worz S, Sander P, Pfannmoller M, Rieker RJ, Joos S, Mechtersheimer G, Boukamp P, Lichter P, Rohr K (2010) 3D geometry-based quantification of colocalizations in multichannel 3D microscopy images of human soft tissue tumors. *IEEE Trans Med Imaging* **29:** 1474-1484
